# Supplementary material for: Conserved principles of central carbon partitioning in Hippo-Yorkie-driven Drosophila gut tumors
Source: bioRxiv. 2026 May 8:2026.05.05.722979. Preprint. [Version 1] doi: 10.64898/2026.05.05.722979 (PMC13174419; doi:10.64898/2026.05.05.722979)
Supplement: 1 [file NIHPP2026.05.05.722979V1-supplement-1.pdf]

bioRxiv preprint doi: https://doi.org/10.64898/2026.05.05.722979; this version posted May 8, 2026. The copyright holder for this preprint (which was not certified by peer review) is the author/funder, who has granted bioRxiv a license to display the preprint in perpetuity. It is made available under aCC-BY 4.0 International license.

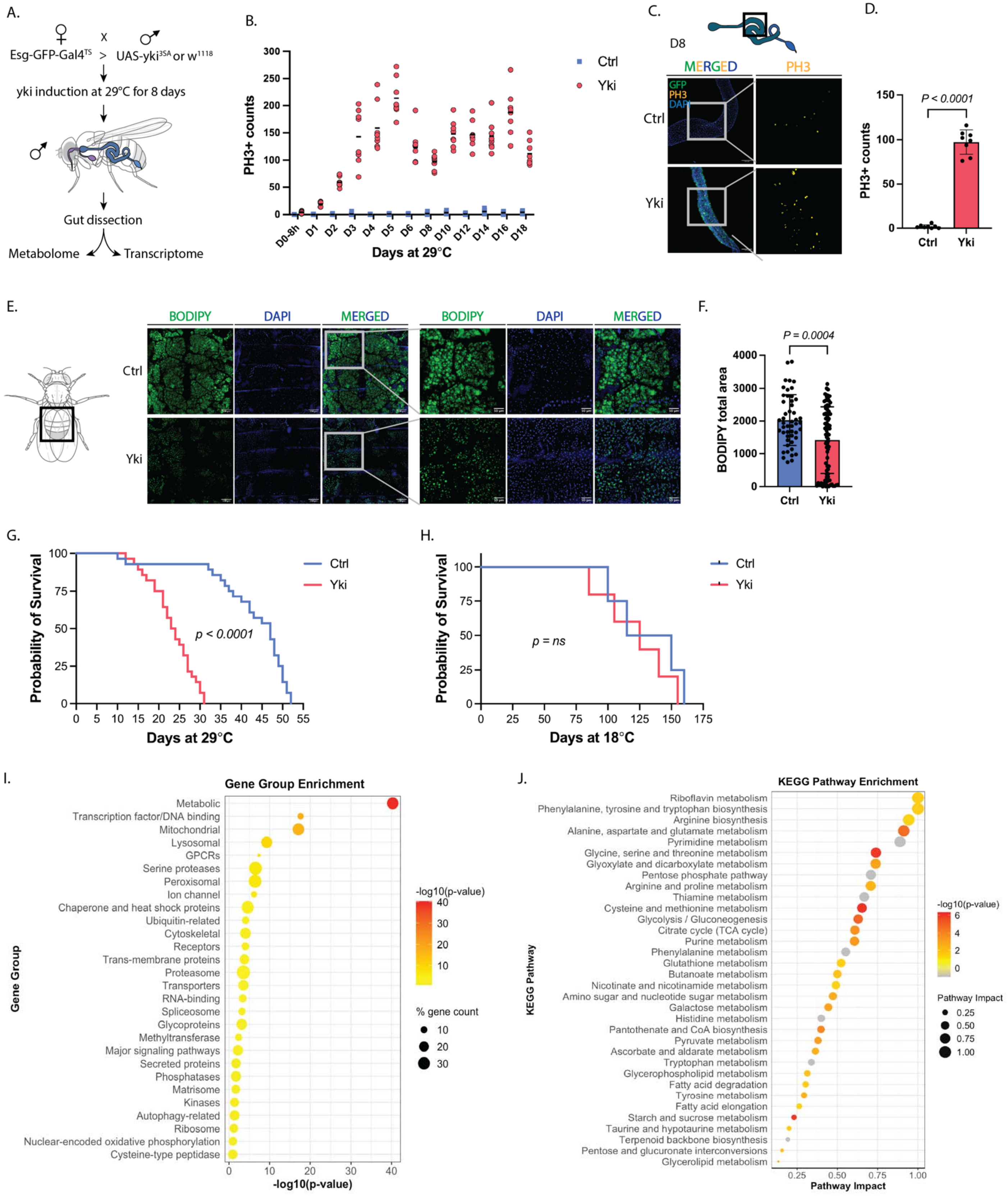

bioRxiv preprint doi: https://doi.org/10.64898/2026.05.05.722979; this version posted May 8, 2026. The copyright holder for this preprint (which was not certified by peer review) is the author/funder, who has granted bioRxiv a license to display the preprint in perpetuity. It is made available under aCC-BY 4.0 International license.

Figure S1. Yki tumors recapitulate organismal hallmarks associated with human cancers.
(A) Experimental schematic for Yki tumor induction and sample collection. *yorkie* (yki^{3SA}) was overexpressed in intestinal stem cells using a temperature-sensitive gene-expression driver; flies were shifted to 29°C for 8 days, followed by gut dissection for metabolome and transcriptome profiling.
(B) Time-course quantification of PH3-positive (PH3+) cells during induction at 29°C demonstrating sustained proliferative activity over time. PH3+ cells were counted in whole Yki guts.
(C-D) Representative images of phospho-histone H3 (PH3) immunostaining in control and Yki-induced midguts at day 8 (C) and quantification of PH3+ cells (D), indicating elevated mitotic activity and increased proliferation in Yki tumors.
(E-F) Representative BODIPY staining of control and Yki tumor-bearing flies (E) and quantification of BODIPY signal (F) showing decreased lipid abundance in Yki flies, consistent with a cachexia-like phenotype.
(G-H) Lifespan analysis at 29°C (G) and under non-inducing control conditions (18°C) (H) showing shortened survival upon yki^{3SA} induction at 29°C, whereas no significant survival difference is observed between genotypes at 18°C.
(I) Gene group enrichment of differentially expressed genes in Yki tumors highlighting significant enrichment of the metabolic gene group.
(J) Pathway enrichment analysis of metabolite profiling data showing broad metabolic remodeling, with prominent alterations in bioenergetic and macromolecule-related pathways, including central carbon metabolism, amino acid metabolism and nucleotide metabolism.

bioRxiv preprint doi: https://doi.org/10.64898/2026.05.05.722979; this version posted May 8, 2026. The copyright holder for this preprint (which was not certified by peer review) is the author/funder, who has granted bioRxiv a license to display the preprint in perpetuity. It is made available under aCC-BY 4.0 International license.

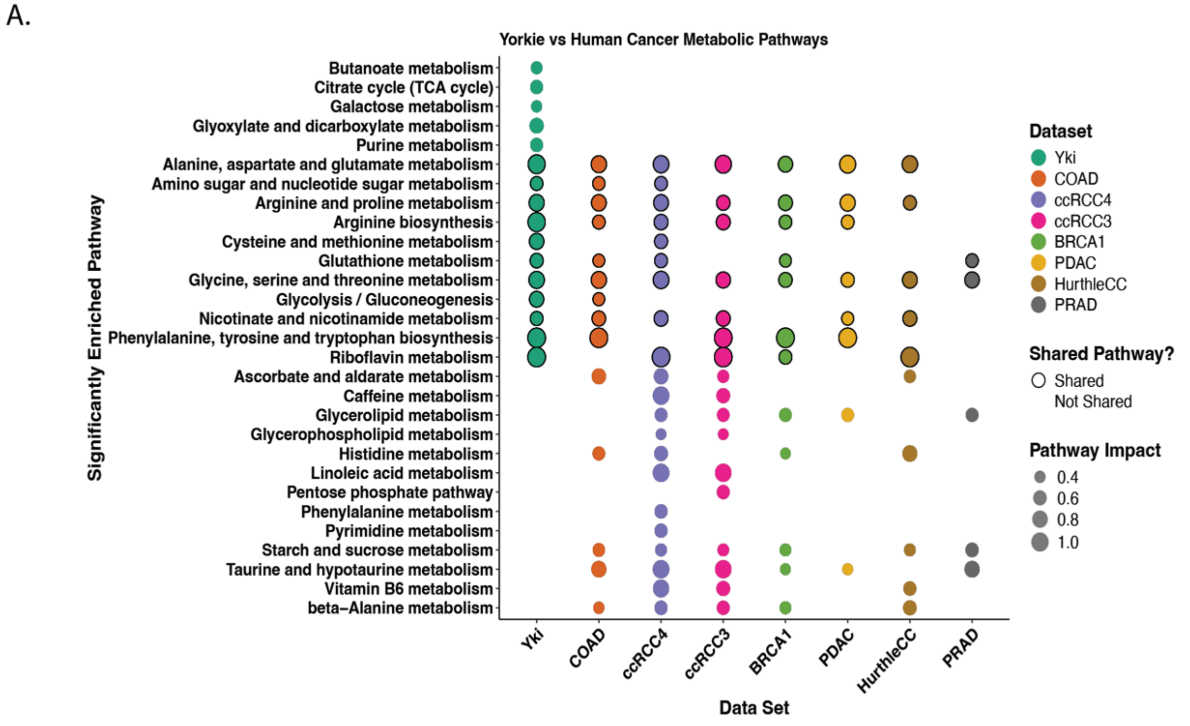

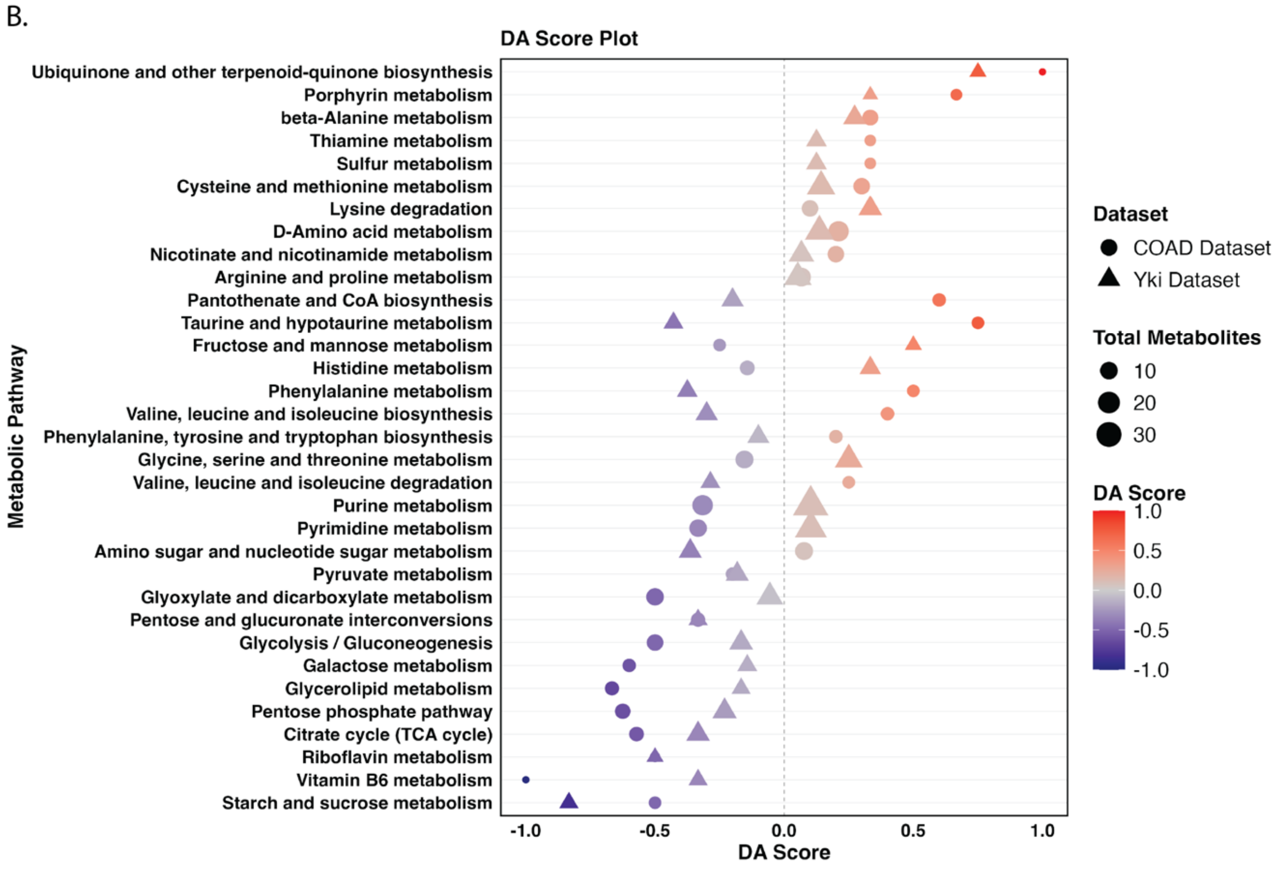

bioRxiv preprint doi: https://doi.org/10.64898/2026.05.05.722979; this version posted May 8, 2026. The copyright holder for this preprint (which was not certified by peer review) is the author/funder, who has granted bioRxiv a license to display the preprint in perpetuity. It is made available under aCC-BY 4.0 International license.

Figure S2. Cross-species comparison reveals shared metabolic pathway rewiring in Yki and human tumors.
(A) Pathway enrichment analysis of Day 8 Yki tumor metabolomics compared with six human cancer types plus one additional subtype. Sixteen KEGG pathways with pathway impact > 0.4 were selected and compared across seven high-quality human cancer metabolomics datasets. Eleven of the 16 Yki-enriched pathways are also enriched in multiple human cancers, with the strongest metabolic overlap observed with human colon cancer (COAD) and clear cell renal cell carcinoma subtype 4 (ccRCC4).
(B) Differential Abundance (DA) analysis comparing Yki tumors and human colon cancer (COAD) reveals concordant up- or downregulation in approximately two-thirds of shared pathways, including glycolysis and amino acid metabolism. The DA score summarizes pathway directionality and is calculated as (number of upregulated metabolites – number of downregulated metabolites)/total number of significant metabolites in the pathway, ranging from +1 (all upregulated) to -1 (all downregulated), with 0 indicating either no significant metabolites or balanced up- and downregulation.

bioRxiv preprint doi: https://doi.org/10.64898/2026.05.05.722979; this version posted May 8, 2026. The copyright holder for this preprint (which was not certified by peer review) is the author/funder, who has granted bioRxiv a license to display the preprint in perpetuity. It is made available under aCC-BY 4.0 International license.

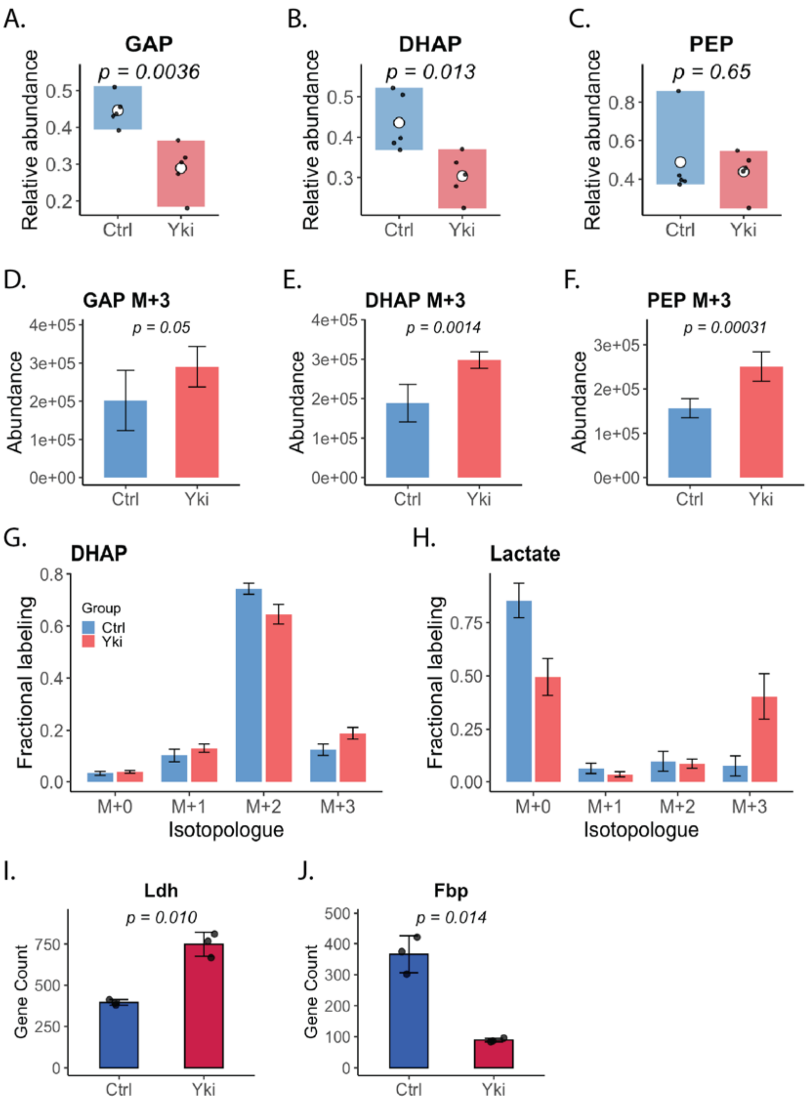

bioRxiv preprint doi: https://doi.org/10.64898/2026.05.05.722979; this version posted May 8, 2026. The copyright holder for this preprint (which was not certified by peer review) is the author/funder, who has granted bioRxiv a license to display the preprint in perpetuity. It is made available under aCC-BY 4.0 International license.

Figure S3 (Related to Figure 1). Expanded metabolic evidence for enhanced glycolytic flux and preferential routing of glucose-derived carbon to lactate in Yki tumors.

(A-C) Relative abundance of the lower-glycolytic intermediates GAP, DHAP and PEP.
(D-F) [U-$^{13}C_6$]glucose tracing showing increased abundance of the fully labeled M+3 isotopologues of GAP, DHAP and PEP in Yki tumors, consistent with enhanced propagation of glucose-derived carbon through lower glycolysis.
(G-H) Fractional labeling of DHAP isotopolgue (M+0-M+3) (G) and lactate (M+0-M+3) (H). Lactate isotopologue fractional labeling shows a decreased in the unlabeled (M+0) fraction and an increased in M+3 lactate in Yki tumors, indicating enhanced incorporation of glucose-derived carbon into lactate.
(I-J) Transcript abundance for *Ldh* (I) and *Fbp* (J), indicating increased expression of lactate dehydrogenase and reduced expression of fructose-bisphosphatase in Yki tumors. Reduced expression of *Fbp* and *Pepck*, which encode rate-limiting enzymes of gluconeogenesis, suggests that glucose-derived carbon flux is preferentially directed downstream through glycolysis rather than diverted into reverse gluconeogenic reactions.

bioRxiv preprint doi: https://doi.org/10.64898/2026.05.05.722979; this version posted May 8, 2026. The copyright holder for this preprint (which was not certified by peer review) is the author/funder, who has granted bioRxiv a license to display the preprint in perpetuity. It is made available under aCC-BY 4.0 International license.

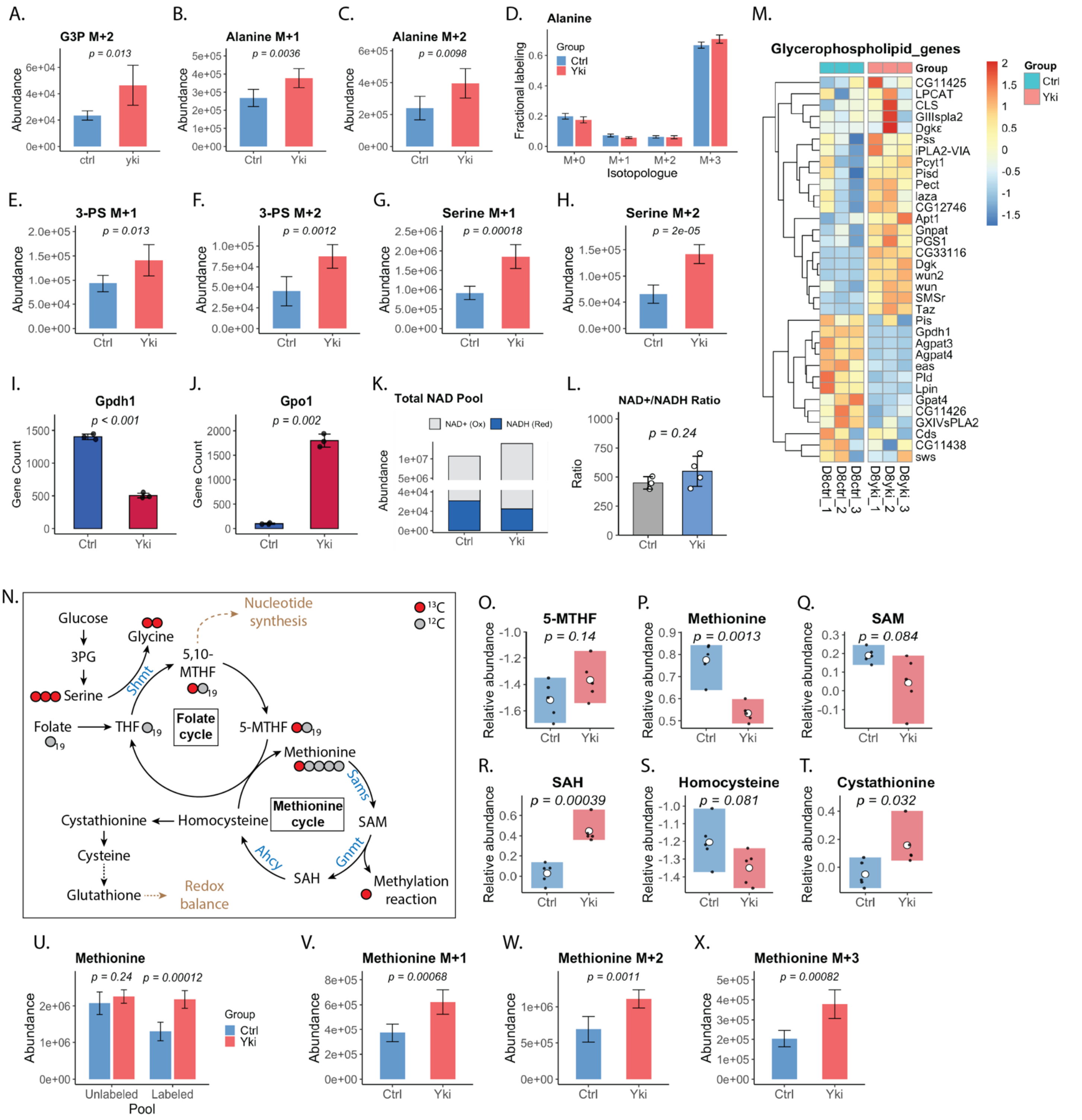

bioRxiv preprint doi: https://doi.org/10.64898/2026.05.05.722979; this version posted May 8, 2026. The copyright holder for this preprint (which was not certified by peer review) is the author/funder, who has granted bioRxiv a license to display the preprint in perpetuity. It is made available under aCC-BY 4.0 International license.

Figure S4 (Related to Figure 2). Expanded analysis of G3P shuttle activity, alanine/serine labeling, redox state, and one-carbon–methionine metabolism in Yki tumors.

(A-C) Abundance of individual istopologues from [U-$^{13}C_6$]glucose tracing in Ctrl and Yki samples. Abundances of G3P M+2 (A), alanine M+1 (B), and M+2 (C).

(D) Fractional labeling of alanine isotopologues (M+0–M+3).

(E-H) Abundances of 3-PS M+1 (E) and M+2 (F), and abundances of serine M+1 (G) and M+2 (H).

(I, J) RNA-seq expression of *Gpdh* (I; cytosolic glycerol 3-phosphate dehydrogenase) and *Gpo1* (J; mitochondrial G3P dehydrogenase) in Ctrl and Yki samples, showing normalized gene counts per sample.

(K) Total NAD pool measured by LC–MS, plotted as stacked abundances of oxidized (NAD^+) and reduced (NADH) species.

(L) NAD^+/NADH ratio in control and Yki samples.

(M) Heatmap of transcripts encoding enzymes involved in glycerophospholipid metabolism.

(N) Schematic of the folate and methionine cycles linking glucose-derived serine to glycine, one-carbon units, methionine, S-adenosylmethionine (SAM), S-adenosylhomocysteine (SAH), and transsulfuration to cystathionine, cysteine, and glutathione; red circles denote potential incorporation of ^13C from [U-$^{13}C_6$]glucose.

(O-T) Relative abundances of 5-methyltetrahydrofolate (5-MTHF; O), methionine (P), SAM (Q), SAH (R), homocysteine (S), and cystathionine (T) in control and Yki samples.

(U) Abundances of unlabeled and total ^{13}C-labeled methionine pools following [U-$^{13}C_6$]glucose tracing.

(V–X) Abundances of methionine M+1 (V), M+2 (W), and M+3 (X) isotopologues, reflecting incorporation of one, two, or three ^{13}C atoms, respectively.

bioRxiv preprint doi: https://doi.org/10.64898/2026.05.05.722979; this version posted May 8, 2026. The copyright holder for this preprint (which was not certified by peer review) is the author/funder, who has granted bioRxiv a license to display the preprint in perpetuity. It is made available under aCC-BY 4.0 International license.

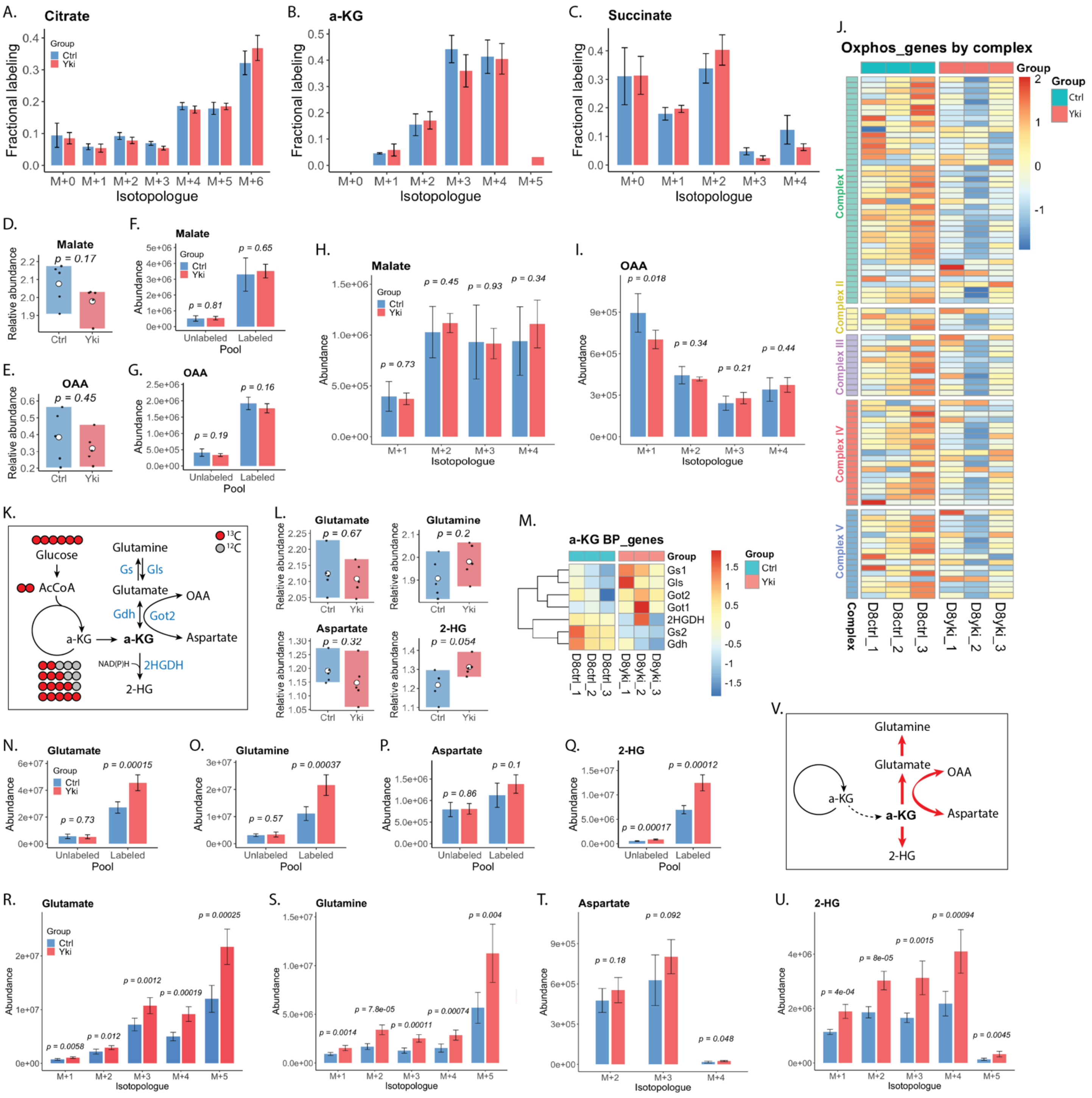

bioRxiv preprint doi: https://doi.org/10.64898/2026.05.05.722979; this version posted May 8, 2026. The copyright holder for this preprint (which was not certified by peer review) is the author/funder, who has granted bioRxiv a license to display the preprint in perpetuity. It is made available under aCC-BY 4.0 International license.

Figure S5 (Related to Figure 3). Glucose-derived carbon is preferentially partitioned at the α-KG node into biosynthetic branches in Yki tumors.

(A–C) Fractional ^{13}C labeling distributions for citrate, α-KG and succinate, highlighting strong labeling in upstream metabolites and an isotopic discontinuity between α-KG and succinate.

(D–G) Additional measurements of downstream TCA-associated intermediates and pools (malate and OAA), including unlabeled versus labeled pool comparisons where indicated.

(H–I) Malate and OAA isotopologue abundances (M+1–M+4), providing further support for preserved glucose contribution to late intermediates despite altered TCA organization.

(J) Heatmap of oxidative phosphorylation (OxPhos) gene expression grouped by respiratory complex, showing coordinated remodeling of mitochondrial bioenergetic programs in Yki tumors. Genes assigned to each complex are listed in the Supplementary Table 1.

(K) Schematic of α-KG branch pathways connecting the TCA cycle to synthesis of glutamate/glutamine, aspartate, and 2-HG.

(L) Relative abundances of α-KG-connected branch metabolites (glutamate, glutamine, aspartate and 2-HG).

(M) Heatmap of transcripts encoding α-KG branch-point enzymes (e.g., aminotransferases, glutamine synthetase and related nodes), supporting increased capacity for α-KG exit flux.

(N–Q) Unlabeled versus ^{13}C-labeled pool abundances for glutamate, glutamine, aspartate and 2-HG, showing increased glucose-derived labeling of α-KG-connected products in Yki tumors.

(R–U) Isotopologue abundances for glutamate, glutamine, aspartate and 2-HG. Notably, M+5 glutamate/glutamine is readily detected and increased in Yki tumors despite undetectable α-KG M+5 in the bulk pool (see panel B in the main figure set), consistent with a rapidly turning-over, highly labeled α-KG sub-pool feeding amino-acid synthesis.

(V) Summary schematic illustrating preferential routing of α-KG-derived carbon into biosynthetic outputs (glutamate/glutamine/aspartate and 2-HG) in Yki tumors.

bioRxiv preprint doi: https://doi.org/10.64898/2026.05.05.722979; this version posted May 8, 2026. The copyright holder for this preprint (which was not certified by peer review) is the author/funder, who has granted bioRxiv a license to display the preprint in perpetuity. It is made available under aCC-BY 4.0 International license.

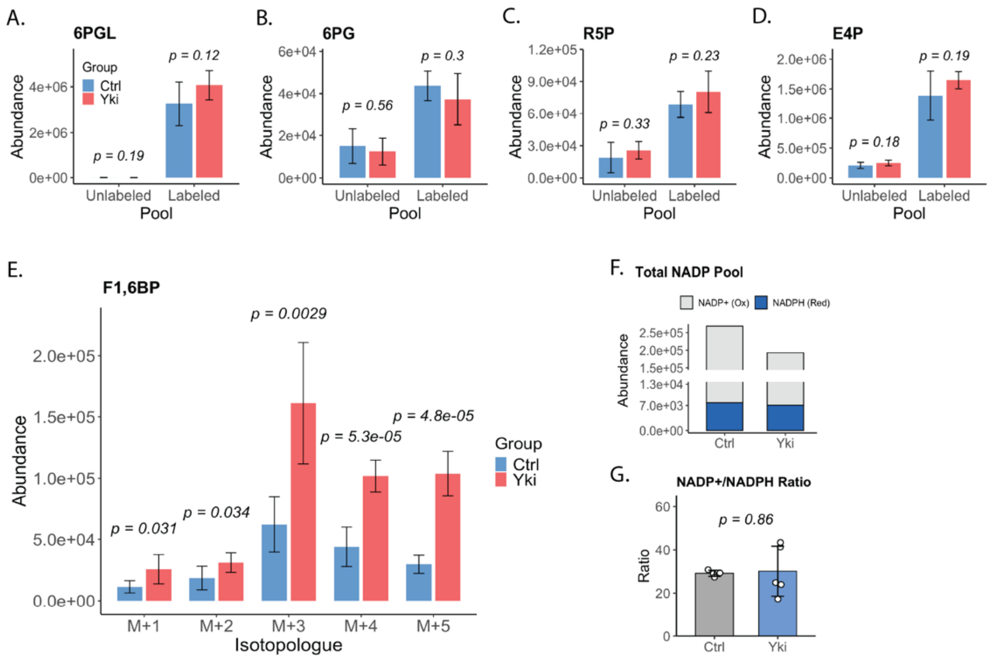

bioRxiv preprint doi: https://doi.org/10.64898/2026.05.05.722979; this version posted May 8, 2026. The copyright holder for this preprint (which was not certified by peer review) is the author/funder, who has granted bioRxiv a license to display the preprint in perpetuity. It is made available under aCC-BY 4.0 International license.

Figure S6 (Related to Figure 4). Expanded metabolic evidence for enhanced non-oxidative PPP activity and preserved NADP(H) redox state in Yki tumors.

(A–C) Abundance of unlabeled and [U-$^{13}C_6$]glucose-derived (labeled) intermediates of the oxidative PPP (oxPPP): 6-phosphogluconolactone (6PGL), 6-phosphogluconate (6PG), and ribose-5-phosphate (R5P). Labeled species dominate each pool, indicating strong glucose contribution, but total labeled + unlabeled pools are not significantly altered in Yki tumors relative to controls.

(D) Abundance of unlabeled and labeled erythrose-4-phosphate (E4P), a non-oxidative PPP (non-oxPPP) intermediate, showing a trend toward increased labeled E4P in Yki tumors.

(E) Abundances of individual fructose-1,6-bisphosphate (F1,6BP) isotopologues (M+1–M+5). Multiple partially labeled species are significantly enriched in Yki tumors, consistent with increased carbon shuffling through the non-oxPPP and reintegration of PPP-derived carbons into upper glycolysis.

(F) Total NADP(H) pool resolved into oxidized NADP+ (grey) and reduced NADPH (blue). Yki tumors exhibit a selective decrease in NADP+ with relatively preserved NADPH levels, indicating a modest contraction of the overall NADP(H) pool while maintaining reducing capacity.

(G) NADP+/NADPH ratio, showing no significant difference between control and Yki tumors, indicating that despite changes in pool size, the NADP(H) redox balance is maintained in Yki tumors.

bioRxiv preprint doi: https://doi.org/10.64898/2026.05.05.722979; this version posted May 8, 2026. The copyright holder for this preprint (which was not certified by peer review) is the author/funder, who has granted bioRxiv a license to display the preprint in perpetuity. It is made available under aCC-BY 4.0 International license.

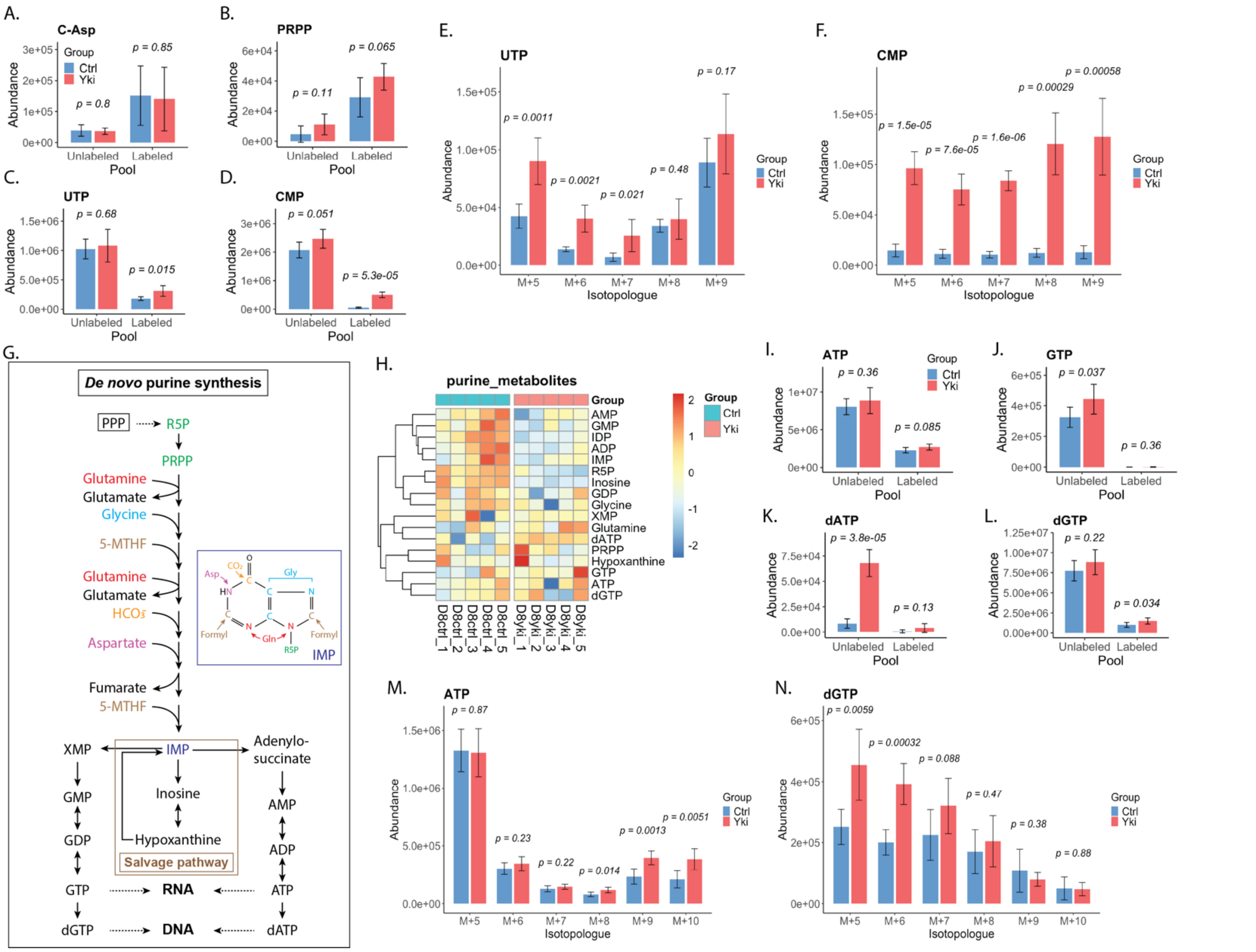

bioRxiv preprint doi: https://doi.org/10.64898/2026.05.05.722979; this version posted May 8, 2026. The copyright holder for this preprint (which was not certified by peer review) is the author/funder, who has granted bioRxiv a license to display the preprint in perpetuity. It is made available under aCC-BY 4.0 International license.

Figure S7 (Related to Figure 5). Glucose-derived carbon supports expanded pyrimidine and purine nucleotide pools in Yki tumors.

(A–D) Abundance of unlabeled and ^{13}C-labeled pools for selected nucleotide precursors and pyrimidine nucleotides (carbamoyl aspartate, PRPP, UTP, and CMP) following [U-$^{13}C_6$]glucose tracing, indicating glucose contribution to nucleotide synthesis.

(E–F) UTP and CMP isotopologue abundances (M+n), showing distribution of glucose-derived labeling across nucleotide carbon backbones.

(G) Schematic of de novo purine synthesis and salvage. Enzymes are shown in blue and metabolites in black; major inputs (e.g., ribose/PRPP, glutamine, glycine, aspartate, bicarbonate/CO_2, and folate-derived one-carbon units) are indicated, with output into RNA- and DNA-directed nucleotide pools.

(H) Heatmap of purine metabolite abundances showing coordinated changes in adenine- and guanine-nucleotide pools in Yki tumors.

(I–L) Abundance of unlabeled and ^{13}C-labeled pools for ATP, GTP, dATP, and dGTP, reporting glucose contribution to ribose-containing purine nucleotides and deoxynucleotides.

(M–N) ATP and dGTP isotopologue abundances (M+n), quantifying incorporation of glucose-derived carbon into purine nucleotide isotopologue pools.

bioRxiv preprint doi: https://doi.org/10.64898/2026.05.05.722979; this version posted May 8, 2026. The copyright holder for this preprint (which was not certified by peer review) is the author/funder, who has granted bioRxiv a license to display the preprint in perpetuity. It is made available under aCC-BY 4.0 International license.

Supplementary Table1. Oxidative phosphorylation (OxPhos) genes grouped by respiratory complex

Complex I	Complex II	Complex III	Complex IV	Complex V
CG40472	SdhA	Cyt-c-p	CG3803	ATPsynB
ND-13A	SdhB	Cyt-c1	COX4	ATPsynC
ND-13B	SdhC	RFeSP	COX4L	ATPsynCF6
ND-15	SdhD	UQCR-11	COX5A	ATPsynD
ND-18		UQCR-11L	COX5B	ATPsynE
ND-19		UQCR-14	COX6A	ATPsynF
ND-20		UQCR-6.4	COX6B	ATPsynG
ND-23		UQCR-C2	COX6C	ATPsynO
ND-24		UQCR-Q	COX7A	ATPsynbeta
ND-30		mt:Cyt-b	COX7AL	ATPsyndelta
ND-39		ox	COX7C	ATPsynepsilonL
ND-42			COX8	ATPsyngamma
ND-49			Cox10	blw
ND-51			Cox11	mt:ATPase6
ND-75			Cox17	mt:ATPase8
ND-ACP			ND-MLRQ	sun
ND-AGGG			mt:CoI	
ND-ASHI			mt:CoII	
ND-B12			mt:CoIII	
ND-B14				
ND-B14.5A				
ND-B14.5B				
ND-B14.7				
ND-B15				
ND-B16.6				
ND-B17				
ND-B17.2				
ND-B18				
ND-B22				
ND-MNLL				
ND-MWFE				
ND-PDSW				
ND-SGDH				
NP15.6				
mt:ND1				
mt:ND2				
mt:ND3				
mt:ND4				
mt:ND4L				
mt:ND5				
mt:ND6				
